# Working conditions in nursing in the face of Covid-19 from the perspective of precariousness

**DOI:** 10.1590/0034-7167-2022-0679

**Published:** 2023-12-04

**Authors:** Ana Paula Nogueira de Magalhães, Diego de Oliveira Souza, Fernanda Pereira de Macêdo, Sabrina Ângela França da Silva Cruz, Camila Pereira-Abagaro, Roselia Arminda Rosales-Flores

**Affiliations:** I Universidade Federal de Alagoas. Arapiraca, Alagoas, Brazil; II Universidad de la Salud. Ciudad de México, México; III Universidade Federal de Pernambuco. Recife, Pernambuco, Brazil; IV Universidad Autónoma de la Ciudad de Mexico. Ciudad de México, Mexico

**Keywords:** Nursing, Nursing, Team, Occupational Health, Working Conditions, COVID-19, Enfermería, Grupo de Enfermería, Salud Laboral, Condiciones de Trabajo, COVID-19, Enfermagem, Equipe de Enfermagem, Saúde do Trabalhador, Condições de Trabalho, COVID-19

## Abstract

**Objective:**

To investigate working conditions in nursing when facing the Covid-19 pandemic, in light of aspects of precarious work.

**Methods:**

Cross-sectional study, with 131 nursing workers who worked against Covid-19 in hospitals in Alagoas State, Northeast Brazil. Data were collected online, using a workers’ health assessment questionnaire. The Chi -Square or Fisher’s Exact test and logistic regression were used.

**Results:**

among nursing workers, 71% had precarious contracts, 33.6% reported extended working hours and 23.7% were union members. In the multivariable analysis, having little hospital experience was a predictor for poor employment (OR= 2.408; 95%CI= 1.051-5.518). The predictor variables for lengthening the working day were being a nurse (OR= 3.824; 95%CI= 1.274-11.483); overtime (OR= 3.668; 95%CI= 1.009-13.333) and inadequate number of workers (OR= 10.872; 95%CI= 3.409-34.675). Being a nursing technician was a predictor of being a union member (OR= 8.967; 95%CI=2.560-31.410).

**Conclusions:**

The pandemic has accentuated the precariousness of working conditions in nursing professionals, especially among nurses.

## RESUMO

**Objetivo:**

Investigar as condições de trabalho em enfermagem no enfrentamento da pandemia de Covid-19, à luz de aspectos da precarização do trabalho.

**Métodos:**

Estudo transversal, com 131 trabalhadoras(es) da enfermagem que atuaram contra a Covid-19 em hospitais alagoanos, no Nordeste brasileiro. Os dados foram coletados online, por questionário de avaliação da saúde dos trabalhadores. Utilizaram-se o teste do Qui-Quadrado ou Exato de Fisher e a regressão logística.

**Resultados:**

Entre as(os) trabalhadoras(es), 71% tiveram contratos precários, 33,6% referiram ao prolongamento da jornada e 23,7% eram sindicalizadas(os). Na análise multivariável, ter pouca experiência hospitalar foi preditor para o vínculo precário (OR= 2,408; IC95%= 1,051-5,518). As variáveis preditoras para o prolongamento da jornada foram: ser enfermeira(o) (OR= 3,824; IC95%= 1,274-11,483), horas extras (OR= 3,668; IC95%=1,009-13,333) e número inadequado de trabalhadoras(es) (OR= 10,872; IC95%= 3,409-34,675). Ser técnica(o) de enfermagem foi preditor para ser sindicalizada(o) (OR= 8,967; IC95%=2,560-31,410).

**Conclusões:**

A pandemia acentuou a precarização das condições de trabalho em enfermagem, principalmente entre as(os) enfermeiras(os).

## RESUMEN

**Objetivo:**

Investigar las condiciones de trabajo de la enfermería para el afrontamiento del Covid-19, según la visión de la precarización del trabajo.

**Métodos:**

Es un estudio transversal realizado entre 131 trabajadoras(es) de enfermería que actuaron contra el Covid-19 en hospitales de Alagoas, Brasil. Los datos se recogieron online mediante un cuestionario de la salud de los trabajadores, con la Prueba X^2^ o Exacto de Fisher y regresión logística.

**Resultados:**

El 71% tenía contratos precarios, 33,6% declaró jornadas laborales prolongadas y 23,7% estaba sindicado. En el análisis multivariado, tener poca experiencia hospitalaria fue un factor predictivo del empleo precario (OR= 2,408; IC95%= 1,051-5,518). Las variables predictoras de jornadas laborales extendidas fueron: ser enfermera(o) (OR= 3,824; IC95%=1,274-11,483); horas extraordinarias (OR= 3,668; IC95%= 1,009-13,333) y número inadecuado de trabajadores (OR= 10,872; IC95% =3,409-34,675). Ser técnica(o) de enfermería fue un factor predictivo de estar sindicado (OR= 8,967; IC 95%= 2,560-31,410).

**Conclusiones:**

La pandemia acentuó la precarización de las condiciones de trabajo en la enfermería, principalmente entre las(os) enfermeras(os).

## INTRODUCTION

The Covid-19 pandemic highlighted the fundamental role of nursing workers in providing care and maintaining people’s lives, promoting a certain visibility and social recognition for workers in this professional field. At the same time, the biggest health crisis in the last 100 years exposed the working conditions to which these workers are subjected, as well as the precariousness of the lives of those who work there^(1)^. Situations such as low pay, job instability, work overload and difficulty in accessing personal protective equipment (PPE)^(1)^ were constant during the pandemic, making these workers vulnerable to physical and mental exhaustion, psychological suffering, illness and death from Covid-19.

In Brazil, at least 4,500 health workers died from Covid-19 in the first two years of the pandemic, of which 70% of the victims were nursing technicians and assistants and 25% were nurses^(2)^. Most deaths occurred among workers who did not have a formal contract and during the period of greatest shortage of personal protective equipment^(2)^.

Historically, working conditions in nursing have been marked by forms of employment designated as atypical, due to the loss of social, union rights and even the weakening of risk prevention involving the work process of these workers. Added to these aspects are intense, long and unregulated working hours; lack of a minimum salary and the pressure for quality and productivity, configuring the nursing work process as something potentially capable of producing illness in its own workers.

Therefore, one of the aspects to be taken into consideration with regard to working conditions in nursing is the precariousness of health work, which can be understood as a complex and multidetermined phenomenon^(3)^, to which several unfavorable characteristics of work accumulate and reinforce each other^(4)^. The term precarious work has come into use in current discourses in order to highlight the working conditions originating from a series of changes that marked the world of work, from the growth of unemployment and the decline in the quality of jobs after productive restructuring, in the second half of the 20th century. The precariousness of work has as its elements the loss of rights, segmentation of workers, weakening of collectives, informalization of work, degradation of health and working conditions, among other aspects^(5)^.

Studies indicate that the phenomenon of precarious working conditions is growing, affecting especially poorer countries and women^(3,6,7)^. However, despite the repercussions of precarious work on the lives of nursing workers, this phenomenon is still little studied, both in national and international research^(3,4,6)^.

In the pandemic situation, few quantitative studies were carried out to better understand nursing working conditions based on the dimensions of precarious work^(4)^ . In view of this, obtaining information about the working conditions of nursing professionals has the potential to equip devices to combat the inequalities experienced since before the pandemic and accentuated by this situation^(1)^.

In view of the above, the following research question arises: What are the working conditions associated with the deepening of precarious nursing jobs during the Covid-19 pandemic? The present study hypothesizes that working conditions in nursing during the Covid-19 pandemic, characterized by precarious employment contracts, extended working hours and low union membership, are associated with other aspects of precarious work, such as the inadequate number of workers, working overtime, having another job, shift rotation and violence suffered in the workplace.

## OBJECTIVE

To investigate working conditions in nursing when facing the Covid-19 pandemic, in light of aspects of precarious work.

## METHODS

### Ethical aspects

The study was approved by the Research Ethics Committee of the *Universidade Federal de Alagoas (UFAL)*, respecting all ethical precepts expressed in Resolutions no. 466/2012 and no. 510/2016, of the National Health Council.

### Study design, period and location

This is an observational, cross-sectional study, carried out from January 1st to June 30th, 2022. The research scenario was made up of field and reference hospitals for serious cases of Covid-19 in the Alagoas municipalities of Maceió, Arapiraca and Santana do Ipanema, Northeast region of Brazil. To prepare the study, the Strengthening recommendations were followed the Reporting of Observational Studies in Epidemiology (STROBE).

### Sample, inclusion and exclusion criteria

Nurses and nursing technicians participated in the study, who provided direct assistance to patients with Covid-19, during the months of April 2020 to April 2021, a period of intense number of cases and deaths in Brazil. The inclusion criterion was having worked in hospital care for patients with Covid-19 for at least one month. Nursing professionals who were removed from work due to being part of the risk group (>60 years old, presence of comorbidities) or who were on vacation at the time of the study data collection were excluded from the study. Based on the eligibility criteria, a non-probabilistic sample was composed, selected by convenience. Of the total 681 nursing professionals invited to participate in the study, 131 responded to the questionnaire, representing a response rate of 19.2%, which is in line with online surveys carried out during the context of the pandemic of Covid-19^(4,8)^. The sample power, calculated later, is 87% and was obtained using the following parameters: n=131, α of 0.05 and effect size of 0.3.

### Study protocol

For data collection, the Individual Questionnaire for the Assessment of Workers’ Health was used, an instrument that is part of the Workers’ Health Assessment and Monitoring Program^(9)^ (Programa de Evaluación y Seguimiento de la Salud de los Trabajadores - PROESSAT), after being translated, adapted and validated by seven Brazilian researchers and one Mexican researcher, with expertise in validating instruments and nursing work processes.

The final version of the adapted instrument presents dichotomous response options (yes/no) and consists of nine sections: 1) Presentation of the instrument, 2) General data, 3) Working conditions, 4) Experience as a nursing worker during the Covid-19 pandemic, 5) Risks and work requirements, 6) Specific questions about the pandemic, 7) Damage to health, 8) Other possible damage and received any medical diagnosis in the last year and, 9) Reflect on how you feel in the present moment.

In this study, questions that make up the section on working conditions (section 3) and the factors that affect them were analyzed. Outcomes were considered precarious contract (yes; no); extending the working day during the pandemic (yes; no) and being a union member (yes; no). These three variables were chosen as outcomes because they consider some of the aspects present in the theoretical debate on the precariousness of work, proposed by Druck^(5)^.

To construct the precarious contract variable, the question about the type of contract during the Covid-19 pandemic was categorized as follows: The answers “fixed-term contract” and “no contract/informal (extra)” were grouped into the category yes for precarious contract and the answers, “single legal regime (public service)” and “CLT (signed contract)”, were considered as no for the variable precarious contract.

The study included sociodemographic variables (gender; age group and skin color), other variables related to working conditions [professional category (nurse); nursing technician); specialization; type of hospital; have another job; overtime; shift rotation; work in the Intensive Care Unit (ICU); extension of the number of hours worked due to illness and/or absence of colleagues; delay in shift change due to patient demands and/or filling out forms; insufficient number of workers for the volume of work and more time to dress and/or undress with personal protective equipment], in addition to variables related to experiences during the pandemic [little hospital experience (<1 year); fear of becoming ill while carrying out work activities; working during the pandemic as a nursing professional had a negative impact on their emotional/family relationships]; to falling ill with Covid-19 (infected by Covid-19 at work) and to the violence suffered as a health worker during the pandemic (violence in the workplace increased during the Covid-19 pandemic; suffered violence from patients and/or their family members; suffered violence from co-workers; suffered violence from superiors/bosses).

Due to the safety protocols necessary to face the Covid-19 pandemic, the Individual Questionnaire for Assessment of Workers’ Health and the Free and Informed Consent Form (TCLE) were inserted into the Google Forms platform and data collection was carried out in online, after consent from the participants. Therefore, the questionnaire was sent to the e-mails of nursing professionals registered in the hospitals. The planned wait time to receive responses was three weeks. After three weeks, a new email was sent as a reminder to the participants.

### Analysis of results and statistics

After collecting and coding the data, an individual analysis of the variables was carried out, using frequency distribution. To identify possible associations between the dependent variables related to working conditions (precarious employment relationship; extension of the number of hours worked during the pandemic and being a union member) and the independent variables, the Chi-Square Test and the Fisher’s Exact Test, adopting a significance of 5%.

The variables that presented statistical associations (p < 0.05) were included in statistical prediction models (binary logistic regression), created for each dependent variable considered in the study: precarious employment relationship; extending the number of hours worked and being a union member. The procedure chosen to select the variables was the forward method stepwise, using the likelihood ratio test. The confidence level adopted in all analyzes was 95%. The Nagelkerke R^2^ value was calculated and to check the fit of the models, the Hosmer and Lemeshow test was used. Data were analyzed using Epi Info™ 7 and SPSS version 25.0.

## RESULTS

Among the 131 study participants, 80.2% (n=105) were women, with a predominance of the age group of 30 to 39 years (41.2%; n=54) and brown skin color (65.6%; n=86). With regard to the categories that make up the professional field, 60.3% (n=79) were nursing technicians and 39.7% (n=52) were nurses. The majority of participants (84.7%; n=111) worked in reference hospitals to treat serious cases of Covid-19, while 15.3% (n=20) worked in field hospitals.

With regard to the working conditions experienced by nursing workers during the Covid-19 pandemic, it was observed that 71% (n=93) of the workers were hired under precarious employment relationships (fixed-term contract or worked without a contract/informal). More than half (54.2%; n=71) reported working in another health service besides the reference/campaign hospital and 64.1% (n=84) reported working overtime.

In addition to working overtime, 74.8% (n=98) worked rotating shifts, working both morning and afternoon or night shifts. In relation to the length of hospital experience, 40.5% (n=53) of nursing workers reported having less than one year of experience, however, almost half of the study participants (49.6%; n=65) worked in ICUs.

Still regarding working conditions, it is observed that the extension of the number of hours worked after the start of the pandemic was pointed out by 33.6% (n=44) of nursing workers. The aspects that led to an increase in the number of hours worked were illness and/or absences of nursing colleagues during the shift (67.9%; n=89); delay in shift change due to patient demands and/or filling out forms (52.7%; n=69); insufficient number of nursing workers for the volume of work (51.1%; n=67) and more time to dress and/ or undress with personal protective equipment (44.3%; n=58). Only 23.7% (n=31) of the nursing professionals were unionized.

It was observed that 80.9% (n=106) of workers reported feeling afraid of falling ill from Covid-19 while carrying out their work activities. Regarding illness, 59.5% (n=78) stated that they were removed from work during the period in which they were on the front line due to falling ill with Covid-19. For 43.5% (n=57) of nursing professionals, working as a nursing had a negative impact on their emotional/family relationships.

Regarding the violence suffered by nursing professionals, 41.2% (n=54) stated that there was an increase in violence in the workplace during the Covid-19 pandemic. Among the study participants, 36.6% (n=48) suffered violence from patients and/or their families, 25.2% (n=33) suffered violence from co-workers and 22.1% (n=29) reported having suffered violence from their bosses or superiors.

Next, the associations between the variables related to working conditions [precarious employment relationship, extended working hours and being a union member] and the independent variables will be presented. In Table 1, it is observed that the precarious employment relationship presented statistical significance with the following variables: having little hospital experience (p=0.049) and fear of becoming ill while working as a nursing worker (p=0.049). In the multivariable analysis, it was observed that having little hospital experience was a predictor for precarious employment contracts (X^2^= 4.605; p<0.05; Nagelkerke’s R^2^=0.049). Workers with little hospital experience (<1 year) were 2.4 times more likely to have a precarious employment relationship during the pandemic (OR= 2.408; 95%CI = 1.051-5.518).

**Table 1:** Variables associated with precarious employment, extended working hours and being a union member among nursing workers in facing the Covid-19 pandemic. Maceió, Arapiraca and Santana do Ipanema, Alagoas, Brazil, 2022

Variables	Precarious work		p	Extension of the working day shift		p	Be a union member		p
	Yes n(%)	No n(%)		Yes n(%)	No n(%)		Yes n(%)	No n(%)	
Professional category			0.439			0.001			<0.001*
Nurse	39(41.9%)	13(34.2%)		26(59.1%)	26(29.9%)		03(9.7%)	49 (49.0%)	
Nursing technician	54(58.1%)	25(65.8%)		18(40.9%)	61(70.1%)		28(90.3%)	51 (51.0%)	
Specialization			0.487			0.015			1.000
Yes	44(62.9%)	19(70.4%)		30(81.1%)	33(55.0%)		14(66.7%)	49 (64.5%)	
No	26(37.1%)	8(29.6%)		7(18.9%)	27(45.0%)		07(33.3%)	27 (35.5%)	
Little hospital experience			0.049			0.573			0.007
Yes	43(46.2%)	10(26.3%)		16(36.4%)	37(42.5%)		06(19.4%)	47 (47.0%)	
No	50(53.8%)	28(73.7%)		28(63.6%)	50(57.5%)		25(80.6%)	53 (53.0%)	
Overtime			0.843			0.000			0.284
Yes	59(63.4%)	25(65.8%)		38(86.4%)	46(52.9%)		17(54.8%)	67 (67.0%)	
No	34(36.6%)	13(34.2%)		06(13.6%)	41(47.1%)		14(45.2%)	33 (33.0%)	
Extension of working hours due to inadequate number of workers			0.248			<0.001			0.685
Yes	51(54.8%)	16(42.1%)		38(86.4%)	29(33.3%)		17(54.8%)	50 (50.0%)	
No	42(45.2%)	22(57.9%)		6(13.6%)	58(66.7%)		14(45.2%)	50 (50.0%)	
Extension of the journey due to longer time to dress and undress with PPE			0.562			0.017			1.000
Yes	43(46.2%)	15(39.5%)		26(59.1%)	32(36.8%)		14(45.2%)	44 (44.0%)	
No	50(53.8%)	23(60.5%)		18(40.9%)	55(63.2%)		17(54.8%)	56 (56.0%)	
Extension of the working day due to delays in shifts			0.448			<0.001			0.839
Yes	51(54.8%)	18(47.4%)		33(75.0%)	36(41.4%)		17(54.8%)	52 (52.0%)	
No	42(45.2%)	20(52.6%)		11(25.0%)	51(58.6%)		14(45.2%)	48 (48.0%)	
Extended working hours due to illness and/or lack of colleagues			0.837			<0.001*			1.000
Yes	64(68.8%)	25(65.8%)		40(90.9%)	49(56.3%)		21(67.7%)	68 (68.0%)	
No	29(31.2%)	13(34.2%)		4(9.1%)	38(43.7%)		10(32.3%)	32 (32.0%)	
Fear of getting sick while working			0.049*			0.640			0.795
Yes	71(76.3%)	35(92.1%)		37(84.1%)	69(79.3%)		26(83.9%)	80 (80.0%)	
No	22(23.7%)	3(7.9%)		7(15.9%)	18(20.7%)		05(16.1%)	20 (20.0%)	
Acquired Covid-19 at work			0.334			0.457			0.150
Yes	49(52.7%)	24(63.2%)		27 (61.4%)	46 (52.9%)		21(67.7%)	52(52.0%)	
No	44(47.3%)	14(36.8%)		17 (38.6%)	41 (47.1%)		10(32.3%)	48(48.0%)	
Work had a negative impact on emotional/ family relationships			0.698			0.005			0.154
Yes	39(41.9%)	18(47.4%)		27(61.4%)	30(34.5%)		17(54.8%)	40 (40.0%)	
No	54(58.1%)	20(52.6%)		17(38.6%)	57(65.5%)		14(45.2%)	60 (60.0%)	
Do you consider that violence in the workplace has increased?			0.241			0.005			0.145
Yes	35(37.6%)	19(50.0%)		26(59.1%)	28(32.2%)		09(29.0%)	45 (45.0%)	
No	58(62.4%)	19(50.0%)		18(40.9%)	59(67.8%)		22(71.0%)	55 (55.0%)	
Suffered violence from patients and/or their families						0.034			0.138
Yes	30(32.3%)	18(47.4%)	0.114	22(50.0%)	26(29.9%)		15(48.4%)	33 (33.0%)	
No	63(67.7%)	20(52.6%)		22(50.0%)	61(70.1%)		16(51.6%)	67 (67.0%)	
Suffered violence from co-workers			0.829			0.018			0.097*
Yes	24(25.8%)	9(23.7%)		17(38.6%)	16(18.4%)		04(12.9%)	29 (29.0%)	
No	69(74.2%)	29(76.3%)		27(61.4%)	71(81.6%)		27(87.1%)	71 (71.0%)	
Suffered violence from superiors/bosses			0.109			0.075			0.014*
Yes	17(18.3%)	12(31.6%)		14(31.8%)	15(17.2%)		02(6.5%)	27 (27.0%)	
No	76(81.7%)	26(68.4%)		30(68.2%)	72(82.8%)		29(93.5%)	73 (73.0%)	

n=number (absolute frequency); p=Value of p from the Chi -Square Test; *P value from Fischer’s exact test.

Regarding the variable prolongation of the number of hours worked due to the pandemic, it is observed that the associated variables were: professional category (p=0.001); specialization (p=0.015); working overtime (p<0.001); increase in the number of hours worked due to the inadequate number of workers (p<0.001); increase in the number of hours worked due to more time spent dressing and undressing with PPE (p=0.017); increase in the number of hours worked due to delays in changing shifts/shifts (p<0.001); increase in the number of hours worked due to illness and/or lack of colleagues (p<0.001); considers that violence in the workplace increased during the Covid-19 pandemic (p=0.005); suffered violence of patients and/or their families (p=0.034) and suffered violence from co-workers (p=0.018).

In the regression model (X^2^ = 39.974; p<0.001; Nagelkerke’s R^2^=0.459), the predictor variables for the extension of the working day were professional category, in which nurses were 3.8 times more likely to have the working day extended (OR= 3.824; 95%CI = 1.274 - 11.483 ); overtime, those who worked overtime were 3.6 times more likely to have their working hours extended (OR = 3.668; 95%CI=1.009 - 13.333) and those who had an increase in the number of hours worked due to the inadequate number of workers, were 10 times more likely to have their working hours extended during the Covid-19 pandemic (OR= 10.872; 95%CI = 3.409 - 34.675).

Regarding the variable being unionized, associations were found with the professional category variables (p<0.001), in which nursing technicians were unionized more frequently than nurses; having little hospital experience (p=0.007), in which unionized women were those who had more hospital experience and suffered violence from bosses/superiors, in which non-unionized women suffered this type of violence is more frequent, as shown in Table 1. When performing the binary logistic regression, it was verified that professional category was the variable that remained in the model (X^2^ = 17.698; p< 0.001; Nagelkerke’s R^2^ = 0.190). Nursing technicians were 8.9 times more likely to be unionized than nurses (OR = 8.967; 95%CI = 2.560 - 31.410).

## DISCUSSION

The results of this research suggest that working conditions in nursing in the face of Covid-19 in Alagoas are inadequate in several aspects, with emphasis on precarious employment relationships, lengthening of the working day, low adherence to unionization, insufficient number of workers in the team, rotating shift work, illness and violence in the workplace. However, it is necessary to emphasize that these problems existed in nursing work before the pandemic, with complex historical roots.

It was observed that the majority of workers who worked to combat the Covid-19 pandemic in Alagoas were hired through precarious employment contracts. The need for a workforce to care for patients with Covid-19 has created many nursing jobs in the country. However, under the support of the Labor Reform in force in Brazil since 2017, entry into these jobs occurred through flexible and temporary contracts^(10)^.

Temporary contracts have been implemented since the 1970s, showing a strong increase in recent years^(11)^. In a Spanish study on the temporary hiring of nurses^(12)^, the authors observed that the proportion of workers with a fixed-term contract grew by 38.7% in 2018. Still in the same study, nurses were the healthcare workers with the highest proportion of temporary contracts in the first four years after completing their degree.

Temporary or informal hiring does not offer security to workers, as it does not guarantee their rights, such as absence from work when health is compromised^(3)^. Given the fragility of the relationship, these workers are more vulnerable to dismissal and, therefore, are subjected to more degrading work situations^(3)^. This reality was evident at the most critical moment of the pandemic, when the fear of losing their jobs led nursing workers to remain in their work activities, even in the absence of appropriate personal protective equipment (PPE) and/or accessible in satisfactory quantities, making them more exposed to illness and death from Covid-19^(10)^.

The workers most vulnerable to precarious contracts are the youngest and those with less training^(7)^, which is in line with the findings of this study, in which having little experience hospital was a predictor of precarious employment. In a study carried out in Rio Grande do Sul, Brazil^(13)^, with the objective of analyzing the health of nursing workers in hospital units dedicated and not dedicated to the treatment of patients with Covid-19, there was also a predominance the precarious work and shorter experience among nursing professionals who worked in units dedicated to patients with Covid-19.

Possibly, the lack of hospital experience, added to the severity of Covid-19 and the insufficient number of personal protective equipment, may have contributed to increasing the fear of these workers of becoming infected or infecting their family members. Furthermore, almost half of the study participants worked in the ICU, a highly complex environment that requires a set of specific skills and in which workers had to constantly deal with critically ill patients and deaths. All these aspects contributed to the deterioration of the physical and mental health of these workers, further increasing precariousness.

In this study, belonging to the nurse category, working overtime and the insufficient number of workers stood out as the main elements related to the extension of the working day. The insufficient number of workers for the adequate dimensioning of human resources in nursing is pointed out in other studies^(1,14)^ as one of the factors that most contribute to the extension of the working day, causing many workers to have to work overtime. Another issue related to the extension of working hours is the low remuneration to which the category is subjected, which leads to a large part of nursing workers to extend their working hours with the accumulation of other employment relationships, an aspect also present among workers in this study.

Several authors demonstrate in their studies the increase in the working hours of nursing workers during the pandemic^(15,16,17,18,19)^. This degrading working condition caused these workers to act in a permanent state of alert, which added to the lack of personal protective equipment^(16)^, reduced time for rest and social interaction with family members and friends, led to exhaustion and physical and mental illness.

Trinkoff et al.^(20)^ had demonstrated the problem of extending working hours in nursing before the pandemic. In a survey carried out in the United States of America (USA), with 2,273 female workers in the area, the authors revealed that more than 25% of the sample worked 12 or more hours a day. When only hospital workers were observed, this percentage exceeded 50%. Among workers with more than one job, the overload was accentuated by the sum of employment, since more than a third worked at least 12 hours a day and almost a quarter worked at least 50 hours a week, without sufficient rest between the shifts.

In the case of Brazil, some data from the research on the nursing profile^(21)^ allow us to have an approximate notion of the national reality of working conditions in the area prior to the pandemic. In the study, with more than 27 thousand participants, a considerable percentage (16.8%) declared that they received R$1,000.00 or less per month. Private and philanthropic institutions were those that most frequently paid the lowest salaries (up to R$1,000.00), which applies to 21.4% of workers in private institutions and 21.5% in philanthropic. Unemployment is a latent problem, considering that 65.9% of workers reported difficulty in finding a job and 10.1% remained unemployed, considering the 12 months prior to the survey.

Regarding the precariousness of nursing working conditions during the pandemic, the study by Llop-Gironés et al.^(11)^ systematized a set of evidence from several countries, which allows us to get a sense of the process internationally. The authors highlight the lack of working conditions with low wages, difficulty in accessing PPE and vaccines, privileged treatment for the medical category, in addition to verbal and physical attacks suffered by nursing workers at work, in the community or even at home.

Rezio et al.^(22)^ confirm the idea of a lack of working conditions during the pandemic, in a study with 719 nursing workers in Brazil. The authors identified the presence of work overload, extended working hours, lack of structure for rest, lack of PPE, low wages, reduction in stable jobs and physical and mental exhaustion. Souza^(23)^ and Rezio et al.^(22)^ locate the roots of this problem in the historical process prior to the pandemic, especially due to the advancement of neoliberal strategies that, in the case of health, have weakened public systems, such as the Unified Health System (SUS), where a large part of the workforce is employed on the front line against Covid-19.

In addition to the inadequate working conditions observed in this study, another aspect found was the lack of political organization, expressed by the low adherence of nursing workers to unions, especially among nurses. Non-unionized nurses more frequently suffered violence from their superiors/bosses and had less hospital experience. Being a union member, therefore, gives significant evidence that it is still a safeguard, at the same time that it contributes to social valorization and, therefore, to the strengthening of the collective of workers itself.

The results of this study also draw attention to the violence suffered in the workplace during the pandemic period. Possibly, inadequate working conditions, associated with the stress of dealing with the severity of the disease for frontline workers, patients and families, led nursing workers to be even more vulnerable to violence, as can be seen in the present study, in which almost half of the participants reported an increase in violence suffered in the workplace, suffering violence from patients and their families, co-workers and bosses. This finding is in line with research carried out in Turkey, where 57.4% of nursing workers stated that during the pandemic there was an increase in the frequency of physical violence and 62.7% reported an increase in the frequency of verbal and psychological violence^(24)^.

In short, given the findings of this research, problems can be highlighted that cover several dimensions of the precariousness of nursing work and that were accentuated in the context of the Covid-19 pandemic. In terms of employment relationships, it was seen that many workers were hired on a temporary basis, just to meet the needs emerging from the pandemic or, what is worse, they already had precarious employment relationships. In relation to the organization and working conditions, changes in the dynamics of work were noted, caused by the lack of knowledge regarding a new disease, the lack of workers, the pressure and responsibility for avoiding deaths in the face of the most tragic event of health in decades and the lack of structure to work. In relation to health, exhaustion, psycho-emotional repercussions and illness due to Covid-19 itself marked the practice of nursing during the pandemic, also in Alagoas. With regard to social recognition and construction of identities, certain stigmas can also be seen within health work, reproducing the biomedical model that places nursing in a subordinate position, as well as remuneration that is not consistent with the label of essential work socially propagated. Finally, on the issue of representation and political organization, the low level of union membership was noted, especially among workers with specialized training/higher level of training, an issue that proved to be as a factor associated with a greater frequency of violence at work.

Given this scenario, the great challenge for nursing workers in Alagoas and, possibly, in Brazil, is to obtain recognition and appreciation for their work from the State and civil society. Those acclaimed as national “heroes”, on the front line of Covid-19, demand to be socially recognized as working women and men worthy of having their rights guaranteed and legitimized. The decisive role of nursing in facing health problems, from the most everyday to historical tragedies, such as the pandemic, makes the need to transform working conditions in nursing urgent. Letting those who are responsible for facing populations’ health problems be the first to lose their own health reveals the contradictory nature of the contemporary world of work and the urgency of understanding it in order to transform it.

### Study Limitations

This study has as one of its limitations the type of non-probabilistic sample, selected for convenience, which hinders the ability to generalize the findings. This strategy was chosen due to social distancing protocols, being widely used to select research participants during the pandemic, through the application of online questionnaires.

Another limitation found was the low response rate, which is one of the main disadvantages observed in studies in which data collection is carried out online^(25)^. Sending reminders to participants’ emails was an attempt to minimize this limitation. However, the congestion of online questionnaires received by e-mail during the pandemic and the work overload experienced by nursing workers may have contributed to the low response rate.

### Contributions to the field of Nursing

The present study contributes to the debate on how precarious work affects nursing, highlighting aspects that characterize the precarious status of nursing work and demonstrating how the Covid-19 pandemic has amplified this problem.

## CONCLUSIONS

The intensification of precarious working conditions in nursing in the context of the Covid-19 pandemic was evidenced in the current research, with emphasis on: 1) precarious employment contracts, of the short-term type, especially among women workers with few years of training and no experience in the ICU environment; 2) excessively long working hours, related to the shortage of nursing workers in the face of pandemic demand, constant illness and multiple work contracts in order to supplement family income and, 3) the low proportion of unionization of nursing professionals, especially nurses, with little hospital experience, leaving them more vulnerable to suffering violence from superiors/bosses.

## Supplementary Material

0034-7167-reben-76-s1-e20220679-suppl01

0034-7167-reben-76-s1-e20220679-suppl02

## Data Availability

https://doi.org/10.48331/scielodata.Q1GE6K
