# Supplementary material for: Working conditions in nursing in the face of Covid-19 from the perspective of precariousness
Source: Rev Bras Enferm. 2023 Dec 4;76(Suppl 1):e20220679. doi: 10.1590/0034-7167-2022-0679 (PMC10695059; doi:10.1590/0034-7167-2022-0679)
Supplement: 0034-7167-reben-76-s1-e20220679-suppl01 [file 0034-7167-reben-76-s1-e20220679-suppl01.pdf]

G	C	P	E	T	A	O	H	R	V	S	P	N	M	A	A	P	A	IN	M	V	V	V	VIOLENCIAAUMENTOU
2	1	1	1	1	1	2	1	1	1	2	2	1	2	2	1	2	2	1	1	2	2	2	1
1	2	1	1	1	1	1	1	1	2	2	1	1	1	2	1	2	1	1	1	2	2	1	1
1	1	1	1	1	1	1	2	1	1	2	2	2	2	2	2	1	2	2	2	2	2	2	2
1	1	1	1	1	2	1	1	1	2	2	1	1	2	2	1	2	1	1	1	1	2	1	1
1	1	1	1	2	2	2	1	2	1	2	1	1	2	2	1	1	1	1	1	1	2	2	1
1	1	1	1	1	2	1	1	1	1	2	2	2	1	1	1	1	2	1	1	2	2	2	2
1	1	1	1	1	2	1	1	2	1	2	1	1	1	1	1	1	2	2	1	2	1	1	2
1	1	1	2	1	2	1	2	2	1	2	2	2	2	2	2	1	2	2	1	2	2	2	1
1	1	1	1	1	1	1	1	1	1	2	1	1	1	1	1	1	2	1	1	1	1	2	1
1	1	1	1	1	1	1	1	1	1	2	1	1	1	2	2	2	2	2	1	2	2	2	1
1	1	1	1	2	2	2	1	1	1	2	2	2	2	2	2	1	1	2	1	1	1	2	1
1	1	1	1	1	2	2	1	2	2	2	1	1	2	1	1	1	1	1	1	1	2	1	1
1	3	1	1	1	1	1	1	1	1	2	2	1	1	1	1	2	1	1	1	2	1	2	2
1	1	1	1	2	1	1	2	1	1	2	1	1	1	1	1	2	2	1	1	2	2	2	1
2	1	1	1	2	2	1	1	2	1	2	1	1	2	2	2	2	1	1	1	2	1	2	2
1	1	1	1	1	2	2	1	1	2	2	1	1	2	1	1	2	1	1	1	1	1	1	1
2	1	1	1	2	2	1	1	1	1	2	2	2	2	2	2	1	1	1	1	1	2	1	1
2	1	1	1	2	2	1	1	1	1	2	2	2	1	2	2	1	1	2	2	2	2	1	2
2	1	1	2	1	1	2	2	2	1	2	2	2	2	2	2	1	1	2	2	2	2	2	2
1	3	1	1	2	2	2	1	1	1	2	2	1	1	1	1	1	1	1	1	1	1	1	1
1	1	1	2	1	1	1	1	2	2	2	1	1	1	1	1	2	1	1	1	1	1	1	2
1	3	1	2	1	1	1	1	2	1	2	1	2	1	1	1	1	1	1	1	1	1	1	1
2	1	1	1	1	1	1	1	2	2	2	1	1	2	1	1	2	2	2	1	1	2	2	1
1	3	1	1	1	2	2	1	1	1	2	2	1	2	1	1	1	2	1	2	2	1	1	1
1	1	1	1	1	1	1	2	1	1	2	2	2	2	2	2	2	2	2	2	2	2	2	2
1	1	1	2	1	1	2	2	2	1	2	2	1	1	1	1	1	1	2	1	1	1	2	1
1	3	1	1	1	2	2	1	1	1	2	1	2	1	1	1	1	1	1	1	2	2	1	1
1	1	1	1	1	2	1	1	2	1	2	1	1	1	1	1	1	2	2	1	2	2	2	2
1	1	1	1	1	1	1	1	1	1	2	1	1	1	1	1	1	1	1	1	1	1	1	1
1	1	1	1	1	2	2	1	1	2	1	1	2	2	2	2	2	1	1	1	1	2	2	2
1	3	1	1	1	1	1	1	1	2	2	2	2	1	1	1	2	1	1	1	1	1	1	1
1	1	1	1	1	2	2	2	2	1	2	2	2	2	2	2	1	2	2	1	2	2	2	2
1	3	1	1	1	2	1	2	1	2	2	2	2	2	1	1	1	2	2	1	2	2	1	1
1	3	2	2	1	2	2	2	1	2	1	2	1	2	1	1	2	1	1	2	1	2	2	1
1	1	2		1	1	2	2	1	1	1	2	1	1	1	1	2	1	2	1	2	2	2	2
2	1	2	2	1	2	1	1	1	1	1	1	1	2	1	1	2	1	1	1	1	2	2	2
1	3	2	1	1	1	1	1	1	1	1	2	1	1	1	1	2	1	2	1	2	2	2	2
1	1	2	1	1	2	2	1	1	1	2	1	1	2	1	1	1	1	1	2	1	1	1	1
2	1	2		1	1	1	2	1	1	2	2	1	1	1	1	2	1	2	1	1	1	1	2
1	1	2	2	1	2	1	2	1	1	2	2	1	1	2	1	2	1	2	1	2	2	2	2
2	1	2		1	1	2	1	1	1	2	1	1	1	1	1	2	1	1	1	2	1	2	1
1	2	2	1	1	1	1	2	1	1	1	1	1	1	1	1	1	1	2	2	1	2	2	2
1	2	2		1	2	2	1	1	2	2	2	1	1	1	1	2	1	1	1	1	1	1	1
1	4	2		1	1	1	1	1	1	2	2	2	2	2	2	1	1	2	2	2	2	2	2
1	1	2	2	1	1	1	1	2	1	1	2	2	2	2	1	2	2	2	1	2	2	2	2
1	3	2	2	1	2	1	2	1	2	2	2	2	1	2	1	2	2	1	1	1	2	2	2
1	3	2	2	1	1	1	1	1	1	2	2	1	2	1	1	1	1	2	1	2	1	1	2
2	1	2	1	1	2	1	1	1	1	2	1	2	1	2	1	1	2	1	1	1	2	2	1
2	2	2	1	1	1	1	2	2	2	2	2	2	2	1	1	2	2	1	1	2	2	2	2
2	1	2	2	1	1	1	1	1	1	2	1	1	1	1	1	2	2	1	1	1	1	2	2
1	1	2		1	1	1	1	1	2	2	2	2	2	1	2	1	1	2	1	1	2	2	2
1	2	2	1	1	1	1	1	1	2	1	1	1	1	1	1	2	1	1	1	1	2	2	1
2	2	2		1	1	2	2	2	1	2	2	2	2	2	2	2	1	1	1	2	2	2	2
1	1	2	2	1	1	1	2	1	2	2	2	2	2	2	2	1	2	2	1	2	2	2	1
1	1	2		1	2	2	1	2	1	2	2	2	2	2	2	1	2	2	2	2	2	2	2
2	1	2	1	2	1	2	1	1	1	1	1	1	1	1	1	2	2	2	1	2	2	2	2
1	4	2	1	2	1	2	1	1	2	1	2	1	1	2	2	2	1	1	1	1	1	2	1

1	3	2	2	2	1	1	1	1	1	2	2	2	2	1	2	2	2	2	1	2	2	2	2
1	3	2	2	2	2	1	2	2	1	2	2	2	2	2	2	1	2	2	2	2	2	2	2
2	1	2		1	1	2	2	2	2	2	2	2	2	2	2	1	2	2	2	2	2	2	2
1	1	2	2	1	2	2	2	1	1	2	2	2	2	1	1	2	2	1	1	2	2	2	1
1	1	2		1	2	1	1	2	2	2	2	2	2	2	2	1	2	2	2	2	2	2	2
2	1	2	1	1	2	1	1	1	1	1	2	1	1	1	1	2	2	1	1	1	1	1	1
1	1	2		1	1	1	2	1	1	2	2	1	2	1	1	2	1	2	1	2	2	2	2
1	1	2	2	1	1	1	1	1	2	2	1	1	1	1	1	2	1	1	1	2	1	1	1
1	1	2		1	1	2	2	1	1	1	2	1	1	1	1	1	1	1	1	2	2	2	2
1	1	2	1	1	2	1	2	1	2	1	2	2	2	2	2	2	1	2	1	2	2	2	2
1	2	2	1	1	1	1	2	1	2	1	2	2	2	2	2	2	1	2	1	2	2	2	2
1	2	2	1	1	1	1	1	1	1	2	2	1	1	1	1	2	2	2	1	2	2	1	2
2	3	2		1	1	2	1	1	1	2	1	1	2	1	1	2	2	1	2	2	1	1	1
1	1	2		1	1	2	1	1	1	2	2	1	2	1	1	2	1	2	2	1	2	2	2
1	1	2	2	1	2	2	1	1	2	2	2	1	1	1	1	2	1	2	1	1	1	1	1
1	3	2	2	1	1	2	1	1	1	2	2	1	2	2	2	2	2	1	2	2	2	2	2
2	1	2		1	1	2	2	1	2	1	2	2	2	2	1	2	1	2	1	2	2	2	2
1	2	2		1	1	2	1	1	1	2	1	1	1	1	1	2	1	1	1	1	1	2	1
1	3	2	2	1	1	2	1	1	1	2	2	1	1	1	1	1	2	2	1	2	2	2	2
1	1	2	2	1	1	2	2	1	1	2	2	1	1	1	1	2	2	1	1	1	1	2	1
2	2	2	2	1	2	1	2	2	2	1	2	2	2	2	1	2	2	2	1	2	2	2	2
2	1	2		1	1	2	2	1	1	1	1	1	2	1	1	2	1	2	2	1	2	2	2
1	1	2		1	1	1	1	1	1	2	1	1	1	1	1	1	2	2	1	2	2	2	2
1	1	2	2	1	2	2	1	2	1	2	2	2	1	1	2	2	2	2	1	2	2	2	2
2	3	2		1	1	2	2	2	1	2	1	1	2	2	1	2	1	2	2	2	2	1	1
1	3	2		1	2	2	1	1	1	2	2	2	1	1	1	1	1	2	1	2	2	2	1
2	3	2		1	1	2	1	1	1	2	2	2	2	2	1	2	2	2	2	2	2	2	2
2	2	2	1	1	2	1	1	1	2	1	1	1	1	1	1	2	1	1	1	1	1	1	1
1	2	2	2	1	1	1	2	1	2	1	2	2	2	2	2	2	2	1	1	1	2	2	2
1	3	1	1	1	1	1	1	2	1	2	2	2	2	2	1	2	2	2	2	1	2	1	1
1	1	1	2	2	1	1	1	1	1	2	1	1	1	1	1	2	1	2	1	1	2	2	1
1	1	1	1	1	1	1	1	1	1	2	1	2	1	1	1	1	1	2	1	2	2	2	2
1	1	1	1	1	1	1	1	2	1	2	1	1	2	2	1	2	2	1	1	2	2	2	2
1	1	2		1	1	2	1	1	2	2	2	2	1	2	1	1	2	2	1	2	2	2	2
1	1	1	1	1	1	2	1	1	1	2	1	1	2	1	1	2	1	2	1	1	2	2	1
1	1	1	1	1	2	1	1	1	2	2	1	1	2	1	1	2	1	2	1	2	2	2	1
1	3	1	1	1	1	1	1	2	1	2	2	2	2	2	2	2	1	2	1	2	2	1	2
1	3	1	1	1	2	1	1	1	2	2	2	2	2	2	2	1	2	2	1	1	1	1	1
1	1	2		1	1	2	1	1	2	2	2	1	1	2	1	2	1	2	1	2	2	2	2
1	1	1	1	1	2	2	1	1	2	2	2	2	1	1	1	1	1	2	1	1	2	2	2
1	1	2		1	2	2	1	1	2	2	1	1	1	1	1	2	2	2	1	2	2	2	2
1	1	2		1	1	2	2	1	2	2	2	2	2	2	1	2	1	2	1	2	2	2	1
1	3	1	1	2	2	1	1	1	1	1	2	1	1	1	1	1	1	1	1	1	2	2	1
1	3	1	1	2	2	1	2	1	1	2	2	2	2	2	2	1	2	2	1	2	2	2	2
1	1	1	1	1	2	2	2	1	2	2	2	2	2	2	2	1	2	2	1	2	2	2	1
1	1	1	1	2	2	1	2	2	1	2	1	2	2	2	1	2	2	2	1	2	2	2	2
1	1	1	1	2	2	2	1	1	1	1	1	1	1	1	1	1	2	2	1	2	2	2	2
1	2	1	1	2	1	2	1	2	1	2	1	1	1	1	1	1	2	1	1	2	1	2	2
1	1	1	1	1	2	2	1	1	1	2	1	1	1	1	1	1	1	2	1	2	1	2	1
1	1	1	1	1	1	2	1	1	1	2	2	2	2	2	1	1	2	1	1	2	2	2	2
1	1	2	1	1	2	1	1	1	2	2	2	2	2	2	2	2	1	2	1	2	2	2	2
2	3	2	1	1	2	2	1	1	1	1	1	1	2	2	2	2	2	1	2	2	2	2	2
1	1	1	1	1	2	2	2	2	1	2	2	2	2	2	2	1	1	2	1	1	2	2	2
1	1	2		2	2	1	1	1	2	1	2	2	2	2	2	2	1	1	1	2	2	2	2
1	1	2	1	1	1	1	1	1	1	2	2	2	2	2	1	2	1	2	1	2	2	2	2
1	4	2		1	1	2	1	1	1	1	2	2	1	1	1	2	1	1	1	1	2	2	1
1	2	2	2	1	2	2	2	1	1	2	2	2	2	2	2	1	2	2	1	2	2	2	2
2	1	2		2	2	1	2	2	1	1	2	1	1	1	1	2	1	2	1	1	2	2	2

1	1	2	2	1	2	1	1	1	1	2	2	2	1	1	1	2	2	2	1	2	2	2	2
1	4	2		1	2	2	1	1	1	1	2	2	2	2	2	1	1	2	1	2	2	2	1
1	1	2	2	1	2	1	1	1	1	2	2	2	2	2	2	2	1	1	1	1	1	2	1
1	3	2	2	2	2	2	2	1	1	1	1	1	2	1	1	2	1	1	2	1	1	2	1
1	1	2		1	2	2	2	1	1	2	2	2	2	2	1	1	2	2	2	2	2	2	2
1	1	2	2	1	2	2	2	1	1	1	2	2	2	2	2	2	1	1	1	2	2	2	2
1	1	1	1	1	2	1	2	2	1	2	2	2	2	2	2	2	1	1	1	2	2	2	2
1	1	2		1	2	1	2	2	1	2	2	1	2	1	1	2	1	2	1	2	2	2	2
1	1	2	1	1	2	2	2	1	1	1	2	2	2	2	1	1	2	1	1	1	2	2	2
2	1	2	2	1	2	1	2	1	1	2	2	1	1	2	1	2	2	2	2	2	2	2	2
1	1	2	2	1	1	2	2	1	1	2	2	2	2	2	2	1	1	2	2	2	2	2	2
1	1	2	2	1	2	1	2	1	1	2	2	1	1	1	1	2	1	1	1	2	2	2	1
1	1	2		1	2	1	1	2	2	1	2	2	1	1	1	2	2	1	1	2	2	2	2
1	1	2		1	2	2	1	1	1	1	2	2	2	2	2	2	2	2	1	2	2	2	2
1	3	2	2	1	1	2	1	1	1	2	2	2	2	2	2	1	1	1	1	1	1	2	1
1	1	2		1	2	1	2	2	1	2	2	1	1	1	1	2	2	2	1	2	2	2	2
