# Supplementary material for: Working conditions in nursing in the face of Covid-19 from the perspective of precariousness
Source: Rev Bras Enferm. 2023 Dec 4;76(Suppl 1):e20220679. doi: 10.1590/0034-7167-2022-0679 (PMC10695059; doi:10.1590/0034-7167-2022-0679)
Supplement: 0034-7167-reben-76-s1-e20220679-suppl02 [file 0034-7167-reben-76-s1-e20220679-suppl02.pdf]

CÓDIGOS DO BANCO DE DADOS - Condições de trabalho da enfermagem no enfrentamento da Covid-19 sob o prisma da precarização

Variáveis sociodemográficas			
**VARIÁVEL**	**DESCRIÇÃO**	**CÓDIGO**	**DESCRIÇÃO DO CÓDIGO**
**FAIXAETARIA**	Idade por faixa etária	**1**	**<20 anos**
		**2**	**20 – 29 anos**
		**3**	**30 – 39 anos**
		**4**	**40 – 49 anos**
		**5**	**50 – 59 anos**
		**6**	**60 anos ou mais**
		**9**	**Ignorado**
**GENERO**	Gênero	**1**<br>**2**<br>**3**<br>**4**	**Feminino**<br>**Masculino**<br>**Outro**<br>**Prefiro não dizer**
**CORPELE**	Cor da pele/etnia autodeclarada	**1**<br>**2**<br>**3**<br>**4**	**Parda**<br>**Preta**<br>**Branca**<br>**Amarela**

Variáveis relacionadas às condições de trabalho			
**VARIÁVEL**	**DESCRIÇÃO**	**CÓDIGO**	**DESCRIÇÃO DO CÓDIGO**
**PROFISSAO**	Diferentes categorias de trabalho em Enfermagem	**1**	**Enfermeira**
		**2**	**Técnica em Enfermagem**
		**3**	**Auxiliar em Enfermagem**
**ESPECIALIZACAO**	Se a trabalhadora(o) tinha especialização (pós-graduação lato sensu)	**1**	**Sim**

		2	**Não**
**TIPODEHOSPITAL**	Tipo de hospital no qual atua ou atuou durante a pandemia de COVID-19:	1	**Hospital de referência**
		2	**Hospital de campanha**
**OUTROTRABALHO**	Se a trabalhadora tinha outro trabalho	1	**Sim**
		2	**Não**
**HORASEXTRAS**	Se trabalhou em regime de horas extras durante a pandemia	1	**Sim**
		2	**Não**
**ROTACAODETURNOS**	Se o profissional de enfermagem trabalhou em escala de rotação de turnos durante a pandemia	1	**Sim**
		2	**Não**
**ATUOUNAUTI**	Se o profissional de enfermagem trabalhou na Unidade de Tratamento Intensivo (UTI) durante a pandemia	1	**Sim**
		2	**Não**
**Principais motivos para o prolongamento da jornada de trabalho:**			
**ADOECFALTACOLEGAS**	Prolongamento da jornada pelo adoecimento e/ou faltas de colegas da enfermagem durante a jornada	1	**Sim**
		2	**Não**
**ATRASOPASSAGEMPLANTAO**	Prolongamento da jornada pelo atraso na passagem de plantão/turno em razão das demandas das/os pacientes e/ou preenchimento de formulários	1	**Sim**
		2	**Não**
**NUMPROINADEQUADO**	Prolongamento da jornada pelo número insuficiente/inadequado de profissionais da enfermagem para o volume de trabalho	1	**Sim**
		2	**Não**
**MAIORTEMPOVESTIRDESPIR**	Prolongamento da jornada pelo maior tempo para vestir-se e/ou despir-se com os equipamentos de proteção individual (batas, aventais ou macacões, luvas, máscaras e equipamentos de respiração/N95-PFF2, óculos de proteção)	1	**Sim**
		2	**Não**

Variáveis dependentes relacionadas às condições de trabalho			
**CONTRATOPRECÁRIO**	Forma de contratação/tipo de contratação para atuar durante a pandemia	**1**	**Contrato por tempo determinado/ sem contrato/informal (extra)**
		**2**	**CLT (carteira assinada) / Regime jurídico único (concursado)**
**SINDICALIZADA**	Se a/o trabalhadora/o está sindicalizada/o	**1**	**Sim**
		**2**	**Não**
**PROLONGAMENTODAJORNADA**	Houve aumento do número de horas trabalhadas após o início da pandemia de COVID-19?	**1**	**Sim**
		**2**	**Não**
**Responda de acordo com sua experiência como enfermeira(o), técnica(o) e/ou auxiliar de enfermagem na pandemia da COVID-19?**			
**POUCAEXPHOSPITALAR**	Se a/o trabalhadora/o apresenta experiência hospitalar menor que um (1) ano	**1**	**Sim**
		**2**	**Não**
**ADQUIRIUCOVID**	Adquiriu Covid-19 no trabalho	**1**	**Sim**
		**2**	**Não**
**IMPACTONEGATIVORELACAO**	O exercício de sua atividade como profissional da enfermagem teve um impacto negativo nas suas relações afetivas/familiares?	**1**	**Sim**
		**2**	**Não**
**MEDOADOECEREXERCICIOPROF**	No início da pandemia (março/abril 2020) você sentiu medo de adoecer no exercício da sua atividade como profissional da enfermagem?	**1**	**Sim**
		**2**	**Não**
**Violência sofrida por ser profissional de saúde no enfrentamento da pandemia**			
**VIOLENCIAAUMENTOU**	Você considera que a violência no ambiente de trabalho aumentou	**1**	**Sim**
		**2**	**Não**

	durante a pandemia de COVID-19?		
**VIOFAMILIARES**	Por parte de pacientes e/ou seus familiares	**1**	**Sim**
		**2**	**Não**
**VIOCOLEGASTRAB**	por parte de colegas de trabalho (enfermeiras, técnicos, médicos etc)	**1**	**Sim**
		**2**	**Não**
**VIOSUPERIORES**	Por parte dos supervisores e ou chefes	**1**	**Sim**
		**2**	**Não**
